# The treatment of continuous double-level lumbar degenerated disease with PE-TLIF: A case report

**DOI:** 10.1097/MD.0000000000041285

**Published:** 2025-01-17

**Authors:** Yufeng Chen, Weiwei Jiang, Xiaoming Ma

**Affiliations:** a Department of Orthopaedic Surgery, Medical College, Yangzhou University, Yangzhou, China; b Department of Orthopaedic Surgery, The Affiliated Taizhou People’s Hospital of Nanjing Medical University, Taizhou, China; c Taizhou School of Clinical Medicine, Nanjing Medical University, Taizhou, China.

**Keywords:** continuous double-level, operative time, PE-TLIF

## Abstract

**Rationale:**

Percutaneous endoscopic transforaminal lumbar interbody fusion (PE-TLIF) is a minimally invasive surgical approach for the treatment of lumbar degenerative diseases, Due to the high incidence of surgical complications and long operative time, the cases reported in previous literature were focused on single segment lumbar degenerative diseases. In our study, we present 2 patients who had continuous double-level lumbar degenerated disease.

**Patient concerns:**

A 71-year-old man with severe low back pain and right leg pain for 3 months, a 57-year-old woman with severe low back pain and bilateral leg pain for 6 months. Conservative treatment is ineffective for both of them.

**Diagnoses:**

Magnetic resonance imaging and physical examination show that the patients had 2 consecutive segments lumbar degenerated disease that required surgical treatment.

**Interventions:**

Considering the poor efficacy of conservative treatment, We performed PE-TLIF on 2 patients.

**Outcomes:**

Finally, both of 2 patients obtained a satisfactory short-term clinical efficacy, without any complications.What’s more, the most crucial thing is that average operative time is only 205 minutes.

**Lessons:**

The patients of continuous double-level lumbar degenerated disease treated with PE-TLIF may provide significant support in selecting the appropriate treatments for similar cases and increase the surgical indications for PE-TLIF.

## 1. Introduction

The percutaneous endoscopic transforaminal lumbar interbody fusion (PE-TLIF) technique was first reported by Osman in 2012.^[1]^ With the development of surgical technique and instrumentation, PE-TLIF was reported to address the variety of spinal disorders by using endoscopic and expandable cages.^[2]^ It allowed directly reaching the disc for achieving simultaneous decompression and fusion without excision of lamina, articular processes, and ligamentum flavum.^[3,4]^ Several literature reported PE-TLIF had advantages of less surgical trauma, less postoperative low-back pain, and faster recovery compared with minimally invasive transforaminal lumbar interbody fusion (MIS-TLIF).^[4–7]^ However, considering the high incidence of surgical complications and long operative time, patients reported in previous papers were single segment involved. In our study, we performed PE-TLIF on 2 patients who had continuous double-level lumbar degenerated disease.

## 2. Case presentation

### 2.1. Case 1

**Figure 1.**
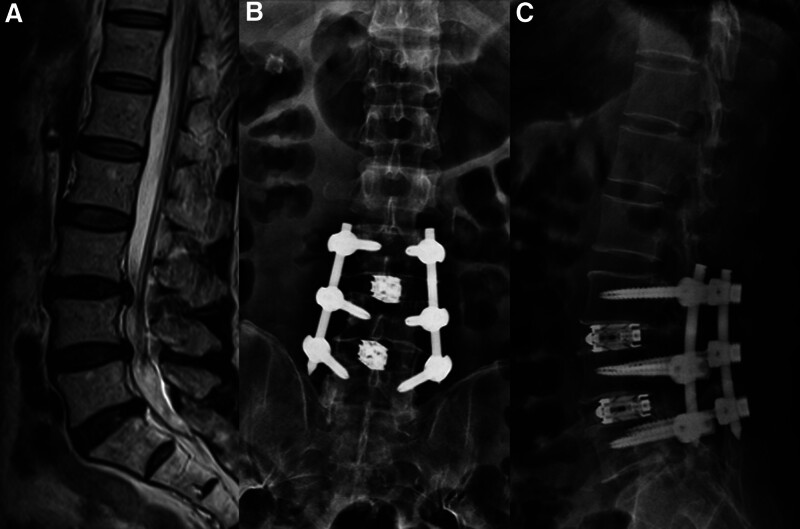
(A) Preoperative MR; (B) and (C) postoperative X-ray. MR = magnetic resonance.

A 71-year-old man was admitted to hospital with severe low back pain and right leg pain for 3 months. The symptoms have not improved after being bedridden and taking nonsteroidal anti-inflammatory drugs (NSAIDs). Magnetic resonance imaging revealed that L3-L5 intervertebral disc herniation (Fig. 1). Subsequently, we performed PE-TLIF on the patient. The operative time was 190 minutes, the blood loss was 100 mL. Six weeks later, the patient obtained a satisfactory clinical efficacy, What’s more, no surgical related complications was observed.

### 2.2. Case 2

**Figure 2.**
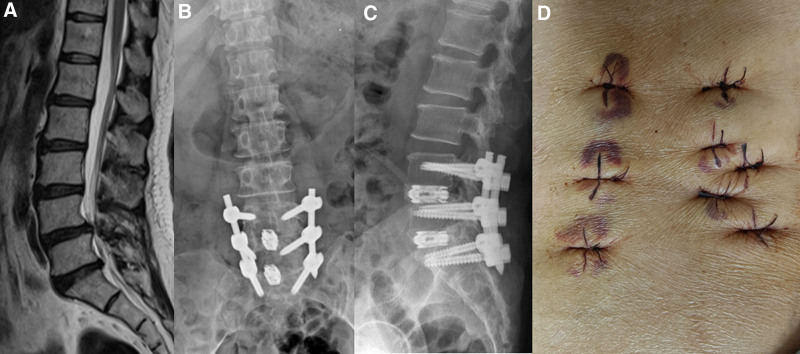
(A) Preoperative MR; (B) and (C) postoperative X-ray; (D) postoperative incision. MR = magnetic resonance.

A 57-year-old woman was admitted to hospital with severe low back pain and bilateral leg pain for 6 months. The symptoms have not improved after being bedridden and taking nonsteroidal anti-inflammatory drugs (NSAIDs) for 3 months. Magnetic resonance imaging revealed that L4 spondylolisthesis combined with L4-S1 intervertebral disc herniation (Fig. 2). Subsequently, we performed PE-TLIF on the patient. Operative time was 220 minutes, the blood loss was 130 mL. Six weeks later, the patient obtained a satisfactory clinical efficacy, What’s more, no surgical related complications was observed.

## 3. Discussion

The incidence of lumbar degenerative diseases is increasing, causing a huge economic burden.^[8]^ Lumbar interbody fusion is the main surgical produce for the treatment of lumbar degenerative diseases and it has been proved to have effective clinical efficacy. However, conventional open surgery may result in greater surgical trauma, as well as increased bleeding and infection risk.^[9,10]^ Sometimes, it can even cause postoperative back pain.^[11]^ Against this drawbacks, minimally invasive surgical procedures are receiving increasing attention in the field of spine surgery.

Foley and colleagues^[12]^ first described the MIS-TLIF technique and it has since become an increasingly popular surgery method for lumbar fusion. MIS-TLIF had the advantages of less blood loss and soft tissue trauma through smaller incision, increase the speed of recovery, and reduce postoperative pain compared with the conventional open technique.^[13]^ However, MIS-TLIF also need to expose paraspinal muscles and excise lamina.

PE-TLIF was first described by Osman in 2012.^[1]^ Jacquot et al reported the incidence of surgical complications is as high as 36% and do not recommend it before technical improvements, although it has many advantages. With the development of surgical technique and instrumentation, PE-TLIF has gained much attention. It can be operated under direct visualization under endoscopy, effectively avoiding damage to nerve roots and dural sac. Several literature had reported PE-TLIF had advantages of less surgical trauma, less postoperative low-back pain, and faster recovery compared with MIS-TLIF.^[4–7]^ However, The locations of the working tube, expandable cage and pedicle screws need to be determined with X-ray during the operation, thus, patients and surgeons are exposed to higher doses of radiation. The learning curve in this technique appeared steep, creating a huge challenge, even for skilled surgeons. The operative time for PE-TLIF is relatively longer and operative time is a risk factor for postoperative complications of lumbar surgery.^[2–4,14]^ Di Capua et al investigated 7761 cases and discovered that operative time ≥ 4 hours is related to postoperative complications.^[15]^

## 4. Conclusion

We firstly reported 2 patients whit continuous double-level lumbar degenerated disease who treated with PE-TLIF. Both of them achieved a satisfactory short-term clinical efficacy and the average operative time is 205 minutes, it is completely acceptable for both patients and surgeons. Thus, we suggested PE-TLIF can effectively relieve the pain and improve the lumbar function of patients with continuous double-level lumbar degenerated disease. It is undeniable that PE-TLIF technique requires a steep learning curve that requiring a thorough understanding of foraminal anatomy and rich endoscopic experience. Due to the limited number of cases reported in this article and the short follow-up time, further research is needed for more cases and longer follow-up time.

Our cases can provide important clues for Surgeons in selecting appropriate treatment in patients with double-level lumbar degenerated disease. PE-TLIF is a feasible and effective surgical technique.

## Author contributions

**Conceptualization:** Xiaoming Ma.

**Data curation:** Yufeng Chen, Weiwei Jiang.

**Formal analysis:** Yufeng Chen, Weiwei Jiang.

**Writing – original draft:** Yufeng Chen, Xiaoming Ma.

**Writing – review & editing:** Yufeng Chen, Xiaoming Ma.
